# Leisure time computer use and overweight development in young adults – a prospective study

**DOI:** 10.1186/s12889-015-2131-5

**Published:** 2015-09-02

**Authors:** Sara Thomée, Lauren Lissner, Mats Hagberg, Anna Grimby-Ekman

**Affiliations:** Occupational and Environmental Medicine, Institute of Medicine, Sahlgrenska Academy, University of Gothenburg, Box 414, 405 30 Gothenburg, Sweden; Epidemiology and Social Medicine, Institute of Medicine, Sahlgrenska Academy, University of Gothenburg, Gothenburg, Sweden

## Abstract

**Background:**

The prevalence of overweight among Swedish young adults has nearly doubled since the 1980s. The weight increase has been paralleled by the increased use of computers at work, at school, and at leisure time. The aim was to examine leisure time computer use for gaming, and for emailing/chatting, in relation to overweight development in young adults.

**Methods:**

A prospective cohort study with Swedish young adults (20–24 years at baseline) who responded to a questionnaire at baseline (*n* = 6735), and after 1 year (*n* = 3928) and 5 years (*n* = 2593). Exposure variables were average daily time spent on leisure time computer gaming and emailing/chatting. Logistic regression was performed for cross-sectional analyses with overweight (BMI ≥ 25) and obesity (BMI ≥ 30) as the outcomes, and for prospective analyses with new cases of overweight at the 1- and 5-year follow-ups. Change in BMI from baseline to 5 year-follow-up was analyzed with linear regression.

**Results:**

There were cross-sectional and prospective associations between computer gaming and overweight (BMI ≥ 25) in women, after adjusting for age, occupation, physical activity, sleep, social support, and total computer use. For the men, only cross-sectional associations could be seen. Spending more than 2 h daily for emailing and chatting was related cross-sectionally to overweight in the women. No clear prospective associations were found for emailing/chatting and overweight development in either sex.

**Conclusions:**

We have identified a new risk group for overweight development: young adult female computer gamers. Leisure time computer gaming was a prospective risk factor for overweight in women even after adjusting for demographic and lifestyle factors, but not in men. There were no clear prospective associations between computer use for emailing/chatting and overweight in either sex.

## Background

The prevalence of overweight among Swedish young adults has nearly doubled since the 1980s [1]. Weight has increased in all age groups, but the increase has been largest among those under 50. This development has been paralleled by the increased use of computers at work, at school and at leisure. In 2013 it was estimated that nearly all 16–24 year old Swedes used computers, and 83 % did so on a daily basis [2]. Social media use on the internet has become a dominating activity. In 2014, 16–25 year olds spent an average of 7.6 h per week on social media (Facebook being the most popular social networking site) [3]. Although women are generally more active in social networking on the internet, there are no major gender differences in how often e.g. email and chatrooms are used [3]. Computer gaming is another dominating leisure time activity. In 2013, about half of Swedish adolescents aged 17–18 played computer or video games at least weekly, and 20 % did so on a daily basis [4]. Computer gaming decreases with age, but there is reason to believe that it will become more and more common among adults as the young gamers grow up to become adult gamers [5]. There are clear gender differences in gaming, with males playing more than females, although female participation in gaming is increasing [6]. For example, the proportion of high consumers of computer games (>3 h daily) was almost 6 times higher among 17–18 year old boys compared to girls (35 % vs 6 %) [4]. The difference was even larger among 13–16 year olds (44 % vs 2 %).

Several studies have shown that screen time is associated with higher BMI or overweight in children and adolescents [7–17]. Time spent on television viewing has often been found to be a risk factor for increased weight (e.g. [8, 12, 15–17]), while there are fewer and less conclusive studies examining computer use. A review by Rey-Lopez et al. [16] found some positive associations between computer use and overweight, but no longitudinal associations that could confirm computer use as a risk factor for weight gain. Falbe et al. [8] found longitudinal associations between digital game playing (computer and video) and BMI increase among girls. In another longitudinal study [13], computer time predicted changes in BMI in boys. Among the cross-sectional studies, Arora et al. [14] found associations between computer use at bedtime and BMI in adolescents, while Kautiainen et al. [12] found associations between time spent on the computer for emailing, writing and surfing the internet and overweight in girls (and similar but non-significant results for boys), but not for time spent on digital games (computer, video or consoles).

There seem to be relatively few studies on the effects of computer use in adult populations. A systematic review of factors associated with sedentary behaviors [18] showed some evidence that screen time, mainly television viewing is related to BMI, even in adults. Only four of the more than 100 included studies examined computer use in this regard and the results were inconclusive; two cross-sectional studies showed a relationship with higher BMI. Furthermore, Vandelanotte et al. [19] found cross-sectional associations between leisure time internet and computer use and overweight and obesity in a study of Australian adults, and, Heinonen et al. [20] found associations between computer use and BMI and waist circumference in women, in a study of Finns aged 30–45.

There is an apparent lack of studies on the effect of computer use on weight in young adults. Young adulthood is a key age to develop and maintain healthy behaviors, as for example overweight in youth tends to follow into adulthood [1, 21]. Hence, it is important for longitudinal studies to examine the potential impact the vast use of computers in this age group may have on overweight development.

### Aims

The aim was to examine the relation between leisure time computer use for gaming and for emailing/chatting with overweight development in young adults.

## Methods

The study design was a prospective cohort study of young adults, with measurements at baseline and after 1 and 5 years. The study was approved by the Regional Ethics Review Board in Gothenburg, Sweden (Registry numbers 191–05 and 876–11).

### Study population and data collection

**Fig. 1:**
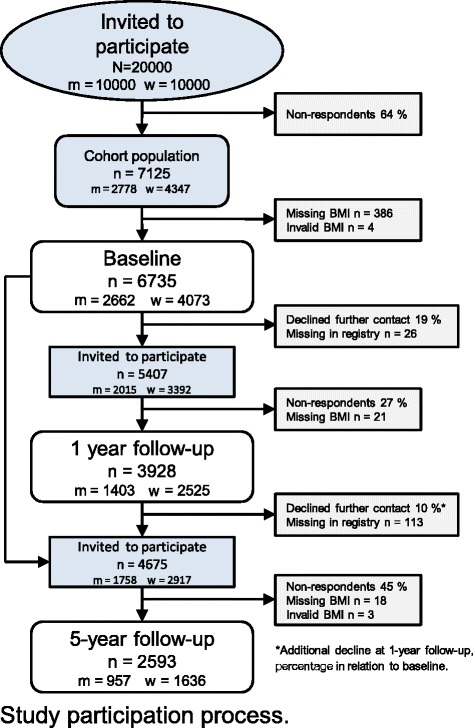
Study participation process. m = men, w = women

A cohort of Swedish young adults aged 20–24 years was recruited in 2007. Twenty thousand persons who were born in the years 1983–1987, 50 % of each sex, were randomly selected from the registry of the total Swedish population (held by the Swedish Tax Agency), and were sent a survey questionnaire containing questions about health, work- and leisure-related exposure factors, demographic factors and lifestyle factors [22]. The questionnaire could be returned by post or completed online, if desired. As an incentive to respond, a lottery ticket (value approx. 1 Euro) was attached to the cover letter and could be used regardless of participation in the study. After two reminders, there were 7125 respondents (36 %). Only those with data on self-reported height and weight, i.e. with data to calculate body mass index (BMI), were included in the current study. An additional four individuals were excluded due to implausible values in height, leaving 2662 men and 4073 women at baseline (Fig. 1). After twelve months, those who had agreed to be invited in future studies (*n* = 5433) were asked by post to respond to an identical web-questionnaire. The data collection process was similar to that at baseline, including the initial lottery ticket, but with the addition of a third reminder offering a paper version of the questionnaire and two cinema tickets for participating. After 3 reminders, the response rate was 73 %, and after exclusion of 21 persons with missing BMI data, *n* = 3928 (1403 men and 2525 women). In 2012, a 5-year follow-up was conducted with an almost identical web-questionnaire. Of 4788 invited (by post and with the lottery ticket incentive), 55 % had responded after three reminders. After excluding three individuals with implausible values for weight and 18 with missing data on BMI, 2593 (957 men and 1636 women) remained in the study (Fig. 1). Of these, 488 were missing at the 1-year follow-up.

### Leisure time computer use

Two aspects of leisure time computer use were examined: computer gaming and emailing/chatting. Self-reported data was collected from the cohort study questionnaire, through the items: a) *On average, how much time per day have you spent on computer gaming (e.g., PC games or online games)?,* and b) *On average, how much time per day have you used on a computer for emailing and chatting?* The items concerned leisure time use the past 30 days. There were four response categories: 1 = *None at all,* 2 = *<1 h per day,* 3 = *1*–*2 h per day,* and 4 = *>2 h per day.* The questions were identical at baseline and at the 1-year follow-up. In the 5-year follow-up questionnaire, the questions were rephrased to also include the use of mobile phones and tablets. In regression analysis of *computer gaming,* response category 1 was used as the reference. In analysis of *emailing/chatting,* response categories 1 and 2 were merged into one and used as the reference, due to the low number of respondents in the lowest category.

### Demographic variables

Demographic information was collected from the baseline questionnaire, including age, highest completed educational level (elementary school, upper secondary school, or college or university studies), and occupation: working, studying, or other (*other* included being on long-term sick leave, or on parental or other leave, or being unemployed). Age and occupation were treated as potential confounders, while educational level was considered to be too closely associated with age in this age group to be included in the models.

### Lifestyle factors

Self-reported leisure time physical activity, sleep duration, social support, and total daily computer use were collected from the baseline questionnaire. Level of leisure time physical activity was measured with a slightly modernized version [23] of the Grimby-Saltin Physical Activity Scale [24, 25]: *How much do you move and exert yourself physically during leisure time? If your activity varies greatly between, for example summer and winter, try to estimate an average. The question concerns thepast 12 months*. There were four response categories: 1 = *physically inactive:* 2 = *light physical activity,* 3 = *regular physical activity,* and *4 = vigorous physical training or competitive sporting*, each containing definitions and examples of activities [23]. In analysis, response categories 3 and 4 were merged into one (*regular or vigorous physical activity*), and thus three categories were used in analyses; *physically inactive, light physical activity* and *regular or vigorous physical activity.* The instrument has been validated in relation to serum cholesterol, blood pressure and BMI [23, 25].

Sleep duration was measured by a question, constructed for the cohort study: *How many hours do you usually sleep per night on…* a) *weekdays (work or study days)?,* and b) *weekends (days off)?* Responses were given in whole hours and concerned the past 30 days. Average sleep duration was calculated ((weekday hours × 5 + weekend hours × 2)/7). Reported sleep values equal or less than 1 h and equal or more than 24 h were considered unreasonable and coded as missing.

The variable *social support* was based on the item: *When I have problems in my private life I have access to support and help*. The item had been constructed for the cohort study as a one-item adaptation of the social support dimension in the Karasek-Theorell model of *demands-control-social support* [26], here relating to private life (rather than working life)*.* Response categories were: 1 = *applies very poorly*; 2 = *applies rather poorly*; 3 = *applies rather well*; 4 = *applies very well.* The responses were categorized as *low* (response categories 1–2), *medium* (response category 3), and *high* (response category 4). The *high* category was used as the reference category.

One questionnaire item was constructed to concern total daily computer use: *On average, how much time per day, have you used a computer? The question concerns total time, i.e., for work, study, and leisure, the past 30 days.* The three response categories were 1 = *<2 h per day*, 2 = *2*–*4 h per day*, and 3 = *>4 h per day.*

A test-retest reliability study and a validating interview study were done in the process of developing the questionnaire. The variables showed moderate to high correlations at test-retest, and the validity was considered acceptable [22].

### BMI outcomes

BMI [kg/m^2^] was calculated based on self-reported height and weight in the cohort questionnaire. Height was included only in the baseline questionnaire, while weight was reported at all three time points. Four implausible heights at baseline were excluded, as well as three unreasonable values for weight (0, 1, 10 kg) at the 5-year follow-up. BMI mean as a continuous variable and BMI categories underweight <18.5, normal range 18.5 – < 25, overweight ≥ 25 – < 30, and obesity ≥ 30*,* are presented in the descriptive statistics*.* The binomial outcome variable *Overweight* was defined as BMI ≥ 25, i.e. including the BMI categories overweight and obesity, and with BMI < 25 as reference. The binomial outcome variable *Obesity* was defined as BMI ≥ 30, with BMI < 30 as reference. The variable *Change in BMI* was calculated as the difference between BMI at the 5-year follow-up and baseline.

### Analysis

All analyses were performed using the SAS statistical package, version 9.3 (SAS Institute, Cary, NC, USA). Descriptive statistics such as means and standard deviations (SD) for continuous variables, and percentages for categorical variables, were calculated with the procedures PROC UNIVARIATE and PROC FREQ, respectively. Descriptive statistics are also shown for the highest category (>2 h per day) of computer gaming and emailing/chatting. Further, leisure time computer use and BMI are presented for the three time points among those who remained at the 5-year follow-up (Table 2).

For all descriptive variables at baseline, sex differences, and differences between the “high gamers” and the rest, as well as between the “high emailers/chatters” and the rest, were analyzed using chi square tests for categorical variables and t-tests for continuous variables. The same tests were used to compare the baseline variables of those who remained in the study at 5-year follow-up and those who did not.

Logistic regression model (PROC LOGISTIC) was used for cross-sectional and prospective analysis. *Overweight* (BMI ≥ 25, i.e. including obesity), and *obesity* (BMI ≥ 30), were outcomes in the cross-sectional analysis of the baseline data. In the prospective analysis, those with BMI ≥ 25 (overweight or obese) at baseline were excluded and the outcomes were new cases of *overweight* (BMI ≥ 25) at the 1- and 5-year follow-ups. Due to a low number of cases, no prospective analyses were done with *obesity* as a separate outcome. First, crude analyses were done with the two primary explanatory variables *gaming* and *emailing/chatting*. Potentially confounding demographic (age, occupation) and lifestyle variables (leisure time physical activity, social support, sleep duration), were adjusted for if p-values ≤0.20 in at least one sex in the univariate analysis. Model I included the demographic variables and in Model II lifestyle factors were added*.* An additional exploratory model was tested by adding total computer time. Only baseline values of the explanatory variables were used.

Spearman correlation analysis showed no collinearity (defined as r >0.7) among the explanatory variables, i.e., gaming, emailing/chatting, total computer use, occupation, age, physical activity, sleep, and social support (the highest correlation was found between emailing/chatting and total computer use; r=0.44).

Supplementary analysis was performed to check the possible influence of partially missing data in the crude and Model I analyses, using the complete cases of Model II.

Furthermore, *change in BMI*, i.e. the difference between BMI at baseline and at 5-year follow-up, was analyzed as the outcome in regression analyses (PROC GENMOD). The same model building as for the logistic regressions was used, with the exception that BMI at baseline was adjusted for in Model Ib.

All analyses were done for men and women separately.

## Results

### Descriptives

At baseline, there were 60 % women and 40 % men (Table 1). Sex differences were observed in all descriptive variables but age. More than half of the males (55 %) reported work as their main occupation, while among the women, an equal proportion worked (43 %) and studied (44 %). Nine and 13 %, respectively, were categorized as “other occupation”, i.e. were unemployed, on sick leave, parental leave, or for other reasons not in work or studies. Mean BMI was 23.7 kg/m^2^ in men, and 22.7 kg/m^2^ in women, and the prevalence of overweight and obesity was 28 % in men and 21 % in women, which corresponds relatively well to the public health statistics from self-reported weights and heights of 16–29 year old Swedes in 2007 [27].

**Table 1:** Study population descriptives

	Baseline study group			Gaming >2 h per day				Emailing/chatting >2 h per day			
	Men	Women		Men		Women		Men		Women	
	%	%	*p* ^*a*^	%	*p* ^*b*^	%	*p* ^*b*^	%	*p* ^*c*^	%	*p* ^*c*^
Education			***		*ns*		*ns*		*ns*		*ns*
Elementary	7	7		10		9		8		7	
Upper secondary	81	78		80		84		78		77	
University	11	15		10		7		14		16	
Occupation			***		***		***		***		*ns*
Working	55	43		48		36		45		41	
Studying	36	44		34		33		38		44	
Other	9	13		18		30		17		15	
*Sick leave*	*1*	*2*		*2*		*5*		*1*		*3*	
*Parental/other leave*	*0*	*4*		*1*		*1*		*1*		*2*	
*Unemployed*	*7*	*7*		*15*		*24*		*14*		*11*	
Computer gaming			***		-		-		***		***
Not at all	47	77		-		-		30		66	
<1 h per day	27	16		-		-		28		21	
1–2 h per day	13	4		-		-		17		7	
>2 h per day	12	2		-		-		25		6	
Emailing/chatting			*		***		***		-		-
Not at all	10	8		8		5		-		-	
<1 h per day	60	63		50		56		-		-	
1–2 h per day	19	21		20		19		-		-	
> 2 h per day	11	9		22		20		-		-	
Total computer use			***		***		***		***		***
< 2 h per day	28	39		2		4		0		2	
2–4 h per day	35	34		19		25		16		24	
> 4 h per day	37	27		78		71		84		74	
Physical activity			***		***		***		**		***
Inactive	17	13		34		40		23		23	
Light	34	46		34		44		36		43	
Regular or vigorous	49	41		33		17		41		35	
Social support			***		*ns*		*		*		***
Low	17	14		16		21		23		21	
Medium	41	32		43		37		39		32	
High	42	55		40		41		38		47	
BMI categories			***		***		**		*		*ns*
Underweight	3	6		5		9		5		6	
Normal	69	73		57		59		64		68	
Overweight	24	16		31		22		27		18	
Obesity	4	5		7		10		4		7	
	Mean (SD)	Mean (SD)	*p* ^*a*^	Mean (SD)	*p* ^*b*^	Mean (SD)	*P* ^*b*^	Mean (SD)	*P* ^*c*^	Mean (SD)	*P* ^*c*^
	*Min-max*	*Min-max*		*Min-max*		*Min-max*		*Min-max*		*Min-max*	
BMI	23.7 (3.4)	22.7 (3.8)	***	24.2 (4.1)	*	24.1 (4.9)	**	23.5 (3.4)	*ns*	23.2 (4.5)	*
	*14.0*–*43.9*	*13.9*–*49.4*		*14.9*–*43.9*		*16.1*–*39.7*		*15.4*–*39.6*		*15.4*–*43.4*	
Sleep duration (hours/night)	7.7 (1.0)	7.8 (1.1)	***	7.7 (1.1)	*ns*	7.9 (1.5)	*ns*	7.8 (1.1)	*ns*	7.6 (1.4)	**
	*3.4*–*13.7*	*2.0*–*13.4*		*3.9*–*12.0*		*4.3*–*12.0*		*3.4*–*12.0*		*3.0*–*12.0*	
Age	22.1 (1.4)	22.0 (1.4)	*ns*	21.9 (1.4)	*	21.8 (1.3)	*ns*	21.7 (1.4)	***	21.7 (1.4)	***
	*20*–*24*	*20*–*24*		*20*–*24*		*20*–*24*		*20*–*24*		*20*–*24*	

The number of subjects varied due to partially missing data. Baseline study group: men *n* = 2612–2662, women *n* = 3966–4073. High gaming: men *n* = 319–325, women *n* = 96–99. High emailing/chatting: men *n* = 272–281, women *n* = 334–344

P-values for differences in the demographic variables; ^a^sex, ^b^high gaming versus the rest, ^c^high emailing/chatting versus the rest. * = *p* < 0.05, ** = *p* < 0.01, *** = *p* < 0.001

Computer gaming was more common among males; more than half of the men played computer games on a daily basis, while a clear majority (77 %) of the females did not. Twenty-five percent of the males spent 1 h or more on computer gaming compared to 6 % of the women. Using the computer for leisure time communication (emailing/chatting) was more common and similar in both sexes, with 30 % of both males and females spending an hour or more on this activity and only about 10 % reported no daily emailing/chatting. About half of the males and 40 % of the females reported that they exercised on a regular basis, while 17 % of the males and 13 % of the females reported being mainly physically inactive at leisure time. Mean reported sleep duration over the week was 7.7 h per night in males and 7.8 in females.

In Table 1, the study demographics are also shown stratified for the highest category (i.e. >2 h) of the leisure time computer variables, and statistical differences between the high groups and the rest can be seen in the table (p-values). The “high gamers” were less often employed or students, compared to the total group. One of four women gamers was unemployed, compared to 7 % in the total group of women, and they more than twice as often reported being on sick leave. Also, the high gaming men as well as the high emailing/chatting men, reported being unemployed about twice as often as the total group of men. The “high gamers” of both sexes less often reported regular physical activity and more often a predominantly physically inactive leisure. The “high gamers” were twice as likely to be “high email/chatters” compared to the total study group, and vice versa. Both “high” groups also reported a total daily computer use of >4 h per day more than twice as often. The high gaming women, and to some degree also the high emailing/chatting women, reported lower social support in private life than the total female study group. Also, among the high emailing/chatting men a slightly lower social support was reported (Table 1).

Furthermore, data on the exposure and outcome variables at the three time points are presented for those who remained in the study from baseline to the 5-year follow-up (*n* = 2593) (Table 2). While the proportion of high gaming males seemed to be steady over the 5-year follow-up period, there was an increase in computer gaming among the women. A decrease of non-gamers can be seen in both men and women. However, the questionnaire item was changed at 5-year follow-up to include also the use of smartphones and tablets, which makes the comparison formally inaccurate. The same applies to the emailing/chatting item. Keeping this in mind, the reported emailing/chatting increased in both groups over the follow-up period; at the 5-year follow-up there were only a few (4 and 1 %, respectively) who reported no daily emailing/chatting. Mean BMI increased about 1 unit over the five years of study in both sexes, and an increase in the prevalence of overweight and obesity can also be seen (Table 2).

**Table 2:** Exposure and outcome variables at baseline, 1-, and 5 year follow-ups

	Men			Women		
	Baseline *n* = 952–957	1 year *n* = 745–749	5 year * n* = 953–957	Baseline * n* = 1622–1636	1 year * n* = 1352–1356	5 year *n* = 1629–1636
Computer gaming %			1)			1)
Not at all	45	46	35	74	75	51
< 1 h per day	29	29	39	18	18	37
1–2 h per day	13	15	16	5	4	7
> 2 h per day	13	10	11	3	3	5
Emailing/chatting %			1)			1)
Not at all	7	5	4	5	3	1
< 1 h per day	62	64	53	66	69	44
1–2 h per day	20	20	27	20	20	37
> 2 h per day	10	11	17	9	8	18
BMI categories %						
Underweight	3	3	2	6	6	3
Normal weight	71	70	61	75	73	70
Overweight	22	23	31	14	15	19
Obesity	4	3	7	5	6	8
BMI Mean (SD)	23.4 (3.3)	23.6 (3.1)	24.5 (3.6)	22.7 (3.7)	22.9 (3.8)	23.7 (4.2)

Respondents that remained at 5-year follow-up. The number of subjects varied due to partially missing data

1) Note: In the 5-year follow-up questionnaire, the gaming and email/chatting items also included the use of mobile phones and tablets, i.e. were no longer restricted to the use of computers

Those who remained in the study at the 5-year follow-up were more likely to be female, students, to have higher educational level, and to report higher computer use and higher physical activity at baseline, compared to those who did not participate after 5 years. The males were also slightly older and had lower BMI at baseline, and the females were slightly more often gamers, compared to those who did not participate after 5 years. No statistical significant different drop-out rates were seen in emailing/chatting, social support or sleep duration at baseline.

### Cross-sectional associations between leisure time computer use and overweight

There were cross-sectional associations between leisure time computer gaming and overweight (BMI ≥ 25) at baseline in both men and women (Table 3). For men, only the highest category (>2 h gaming per day) had a clear association with overweight in the crude analysis (OR 1.7), but after adjusting for demographic and lifestyle factors in Models I and II, also the next-to-highest category (1–2 h gaming per day) was associated with increased overweight (OR 1.4, Model II). For women, all the gaming categories were associated with overweight (ORs 1.6–2.2, Model II, Table 3).

**Table 3:** Cross-sectional logistic regressions for leisure time computer use and overweight at baseline

		Crude			Model I		Model II	
		OW %	OR	95 % CI	OR	95 % CI	OR	95 % CI
GAMING								
Men	>2 h	38	**1.7**	**1.30–2.17**	**1.8**	**1.38–2.36**	**1.7**	**1.30–2.26**
	1–2 h	30	1.2	0.93–1.56	**1.3**	**1.01–1.73**	**1.4**	**1.03–1.78**
	<1 h	26	1.0	0.79–1.19	1.0	0.81–1.24	1.0	0.80–1.24
	Not at all	27	1.0		1.0		1.0	
Women	>2 h	32	**2.2**	**1.42–3.37**	**2.0**	**1.29–3.16**	**1.7**	**1.06–2.74**
	1–2 h	37	**2.6**	**1.92–3.65**	**2.5**	**1.76–3.46**	**2.2**	**1.56–3.17**
	<1 h	26	**1.6**	**1.32–1.96**	**1.6**	**1.27–1.91**	**1.6**	**1.28–1.95**
	Not at all	18	1.0		1.0		1.0	
EMAILING/CHATTING								
Men	>2 h	31	1.1	0.84–1.44	1.2	0.89–1.56	1.1	0.84–1.50
	1–2 h	24	**0.8**	**0.63–0.98**	0.9	0.68–1.07	0.9	0.68–1.10
	<1 h or 0	29	1.0		1.0		1.0	
Women	>2 h	26	**1.4**	**1.05–1.77**	**1.4**	**1.10–1.89**	**1.4**	**1.05–1.83**
	1–2 h	20	1.0	0.81–1.20	1.1	0.87–1.30	1.0	0.85–1.28
	<1 h or 0	20	1.0		1.0		1.0	

OW % = prevalence of overweight. Odds ratios (OR) are presented with 95 % confidence intervals (CI). MODEL I: Adjusted for age, occupation, MODEL II: age, occupation, physical activity, social support, sleep duration, The number of subjects varied in the models due to partially missing data. GAMING: men *n* = 2515–2647, women *n* = 3806–4052. EMAIL/CHAT: men *n* = 2512–2643, women *n* = 3797-4039. ORs with a CI not including 1.00 are given in bold

Leisure time emailing and chatting >2 h per day was associated with overweight in the women (OR 1.4, Model II, Table 3), but not in the men. For the men, medium (1–2 h daily) emailing or chatting was actually associated with a lower prevalence of overweight in the crude analysis (OR 0.8). However, when adjusting for demographic and lifestyle factors the negative association was no longer statistically significant.

A supplementary complete case analysis was performed to check possible influence of partially missing data in the crude and Model 1 analyses. No major effects on results were seen except for the loss of statistical significance of medium emailing/chatting in the men in the crude analysis. All in all, the ORs changed in the range of −0.2 to +0.1, and there were slight changes in the confidence limits, which in most cases meant a widening of the CIs.

An additional, exploratory third model, which included total time spent on computer, was tested, and seemed to strengthen the existing associations over all (data not shown).

### Cross-sectional associations between leisure time computer use and obesity

The patterns when cross-sectionally analyzing *obesity* (BMI ≥ 30) were similar to the above analyses of *overweight*. There were clear associations between computer gaming and obesity in both sexes (data not shown in table). For the men, the associations were amplified, compared to overweight being the outcome; the two highest categories of gaming (>2 h and 1–2 h) generated ORs 1.8 (CI 1.02–3.04) and 2.0 (CI 1.17–3.38) after adjusting for demographic and lifestyle factors. For the women, the three gaming categories (>2 h, 1–2 h, <1 h) generated ORs 2.1 (CI 1.02–4.47), 2.9 (CI 1.64–4.96), and 2.2 (CI 1.54–3.15) in Model II. No clear associations were found between leisure time emailing/chatting and obesity in either sex. For the men, high and medium emailing/chatting generated ORs of 0.7 (CI 0.34–1.36) and 0.8 (CI 0.49–1.41) and for the women 1.5 (CI 0.95–2.47) and 1.1 (CI 0.75–1.62) in Model II.

### Prospective associations between leisure time computer use and overweight

In the prospective analyses, i.e. after excluding those with BMI ≥ 25 at baseline, high computer gaming (>2 h per day) at baseline meant higher odds for overweight at 1-year follow-up for the women (OR 3.2, Model II) (Table 4). Furthermore, the two highest categories of gaming (>2 h and 1–2 h) at baseline, were steadily associated with new cases of overweight at 5-year follow-up (ORs 3.0 and 2.7, Model II). The additional, third model which adjusted for total daily computer use, slightly amplified the associations. For the men, no statistically significant prospective associations were seen between computer gaming and overweight at either follow-up. Moreover, no statistically significant prospective associations between leisure time emailing and chatting and overweight were seen in either sex (Table 4). Complete case crude and Model I analyses did not change results notably. Due to a low number of cases, no prospective analyses were done with obesity (BMI ≥ 30) as a separate outcome.

**Table 4:** Prospective logistic regressions for leisure time computer use at baseline and new cases of overweight at 1- and 5-year follow-ups

		1-YEAR							5-YEAR						
			Crude		Model I		Model II			Crude		Model I		Model II	
		OW %	OR	95 % CI	OR	95 % CI			OW %	OR	95 % CI	OR	95 % CI	OR	95 % CI
GAMING															
Men	>2 h	9	1.2	0.54–2.69	1.2	0.55–2.78	1.2	0.53–2.78	28	1.5	0.86–2.68	1.6	0.88–2.84	1.4	0.77–2.66
	1–2 h	6	0.8	0.36–1.77	0.8	0.37–1.82	0.8	0.36–1.81	18	0.9	0.49–1.64	1.0	0.52–1.79	0.9	0.48–1.69
	<1 h	9	1.3	0.78–2.21	1.3	0.76–2.19	1.3	0.74–2.14	19	0.9	0.60–1.43	0.9	0.60–1.47	0.9	0.58–1.42
	Not at all	7	1.0		1.0		1.0		20	1.0		1.0			
Women	>2 h	15	**3.4**	**1.38–8.26**	**2.8**	**1.13–7.14**	**3.2**	**1.23–8.12**	31	**3.2**	**1.41–7.10**	**3.0**	**1.33–6.82**	**3.0**	**1.29–6.83**
	1–2 h	9	2.0	0.89–4.49	1.6	0.66–3.74	1.7	0.71–4.10	32	**3.3**	**1.83–5.78**	**2.8**	**1.54–5.11**	**2.7**	**1.45–5.01**
	<1 h	7	1.5	0.91–2.38	1.4	0.88–2.32	1.4	0.86–2.30	13	1.1	0.68–1.63	1.0	0.67–1.62	1.0	0.63–1.56
	Not at all	5	1.0		1.0		1.0		12	1.0		1.0		1.0	
EMAILING/CHATTING															
Men	>2 h	10	1.5	0.74–3.11	1.6	0.77–3.30	1.6	0.78–3.37	25	1.4	0.79–2.60	1.5	0.80–2.67	1.5	0.81–2.72
	1–2 h	9	1.3	0.74–2.28	1.4	0.77–2.39	1.3	0.72–2.29	22	1.2	0.75–1.84	1.2	0.77–1.90	1.2	0.77–1.94
	<1 h or 0	7	1.0		1.0		1.0		19	1.0		1.0		1.0	
Women	>2 h	7	1.3	0.69–2.54	1.3	0.67–2.52	1.4	0.69–2.66	16	1.2	0.69–2.01	1.2	0.70–2.05	1.2	0.69–2.05
	1–2 h	7	1.3	0.84–2.01	1.4	0.88–2.17	1.5	0.93–2.33	11	0.8	0.51–1.18	0.8	0.53–1.24	0.8	0.54–1.28
	<1 h or 0	5	1.0		1.0		1.0		14	1.0		1.0		1.0	

OW % = prevalence of new cases of overweight at follow-ups. Odds ratios (OR) are presented with 95 % confidence intervals (CI). 1-YEAR MODEL I is adjusted for age and occupation, 1-YEAR MODEL II: age, occupation, physical activity, social support, sleep duration. 5-YEAR MODEL I is adjusted for occupation, 5-YEAR MODEL II: occupation, physical activity. The number of subjects varied in the models due to partially missing data. 1-YEAR GAMING: men *n* = 979–1013 women *n* = 1940–2023. 1-YEAR EMAILING/CHATTING: men *n* = 979–1013, women *n* = 1937–2019. 5-YEAR GAMING: men *n* = 691–703, women *n* = 1269–1315. 5-YEAR EMAILING/CHATTING: men *n* = 691–703, women *n* = 1265-1310. ORs with a CI not including 1.00 are given in bold

### Change in BMI from baseline to 5-year follow-up

Linear regressions with change in BMI from baseline to 5-year follow-up as the outcome showed a higher BMI-increase for all computer gaming categories in all the models for the women (Table 5). A dose–response relationship emerged, however, with overlapping CIs in most analyses. Belonging to the highest category of female gamers implied an additional BMI-increase of an estimated 1.33 BMI units (Model II), while even gaming less than 1 h per day implied an estimated increase of 0.51 BMI units (Model II). The estimated total increase in BMI from baseline to 5-year follow-up in the female group, i.e. when also taking the “natural” change in BMI into account, was 0.79 BMI-units in no-gamers, 1.30 in <1 h gamers, 1.66 in 1–2 h gamers, and 2.12 in >2 h gamers. No clear associations were seen between change in BMI and computer gaming for men or for emailing/chatting in either sex (Table 5). Complete case crude and Model I analyses did not change results notably.

**Table 5:** Linear regressions for leisure time computer use at baseline and change in BMI from baseline to 5-year follow-up

		Crude		Model Ia		Model 1b		Model II	
		Estimate	95 % CI	Estimate	95 % CI	Estimate	95 % CI	Estimate	95 % CI
GAMING									
Men	>2 h	0.19	−0.23; 0.61	0.19	−0.23; 0.61	0.29	−0.13; 0.70	0.19	−0.24; 0.62
	1–2 h	0.08	−0.34; 0.49	0.07	−0.34; 0.49	0.14	−0.27; 0.55	0.12	−0.30; 0.54
	<1 h	−0.03	−0.35; 0.29	−0.04	−0.36; 0.28	−0.01	−0.32; 0.30	−0.06	−0.33; 0.26
	Not at all	0.00				0.00		0.00	
Women	>2 h	**1.07**	**0.30; 1.84**	**1.04**	**0.27; 1.82**	**1.30**	**0.54; 2.07**	**1.33**	**0.56; 2.10**
	1–2 h	**0.81**	**0.25; 1.37**	**0.79**	**0.23; 1.35**	**0.96**	**0.40; 1.51**	**0.86**	**0.29; 1.43**
	<1 h	**0.43**	**0.10; 0.76**	**0.42**	**0.09; 0.75**	**0.53**	**0.20; 0.85**	**0.51**	**0.18; 0.84**
	Not at all	0.00		0.00		0.00		0.00	
EMAILING/CHATTING									
Men	>2 h	0.07	−0.38; 0.51	0.05	−0.40; 0.49	0.01	−0.43; 0.44	−0.02	−0.46; 0.43
	1–2 h	−0.07	−0.41; 0.27	−0.08	−0.42; 0.25	−0.09	−0.43; 0.24	−0.10	−0.43; 0.24
	<1 h or 0	0.00		0.00		0.00		0.00	
Women	>2 h	−0.18	−0.62; 0.26	−0.20	−0.64; 0.24	−0.14	−0.57; 0.30	−0.17	−0.61; 0.27
	1–2 h	0.05	−0.26; 0.37	0.05	−0.27; 0.36	0.06	−0.26; 0.37	0.12	−0.19; 0.44
	<1 h or 0	0.00		0.00		0.00		0.00	

Parameter estimates from the linear regressions with 95 % confidence intervals (CI) are presented. MODEL Ia is adjusted for age, MODEL Ib: age, BMI, MODEL II: age, BMI, physical activity, social support. The number of subjects varied in the models due to partially missing data. GAMING: men *n* = 952–937, women *n* = 1631–1592. EMAILING/CHATTING: men *n* = 952–937, women *n* = 1622-1586. Estimates with a CI not including 0.00 are given in bold

Because adjusting for baseline BMI (Model 1b) showed an increase in the estimated change in BMI, we also split the dataset and performed analyses in the subsets of those with BMI ≥ 25 and those with BMI < 25. The increased change in BMI was only statistically significant in the females with BMI < 25 (data not shown).

## Discussion

There were clear cross-sectional and prospective associations between computer gaming and overweight (BMI ≥ 25) among the young women in this study, even after adjusting for demographic (age, occupation) and life style (physical activity, sleep, social support, total computer time) factors. For the young men, only cross-sectional associations could be detected. Leisure time computer use for communicating was not to the same extent related to overweight; spending more than 2 h daily for emailing and chatting was cross-sectionally related to overweight only among the women. No clear prospective associations were found for emailing/chatting and overweight or increased change in BMI in either sex.

The results are partly in line with earlier studies finding a relationship between screen time and overweight or BMI in children and adults (e.g. [7–20, 28]). However, to our knowledge there are only few studies that have specifically examined computer use as a risk factor for overweight in adults, and only cross-sectional associations seem to have been found previously [18–20]. The present study suggests that the content of the computer use can be of importance, as time spent on computer gaming appeared to be more connected to weight gain than time spent on emailing or chatting. In this regard, Kautiainen et al. [12] found associations between time spent on the computer for emailing, writing and surfing the internet and overweight, but not for time spent on digital games, which is partly inconsistent with our study. However, the Kautiainen study was cross-sectional and in a younger age group (14–18 years). That the increased change in BMI from baseline to 5-year follow-up in relation to computer gaming was seen mainly among the normal weight women and not among the overweight, is contrary to the results of Falbe et al. [8] and Mitchell et al. [9], where associations were stronger among the overweight.

The prospective results seen only in the women in the present study possibly indicate gender differences. In the study of Finnish adults, Heinonen et al. [20] found cross-sectional associations between computer time and BMI and waist circumference in the females only. Gender differences have also been found in some of the studies on children or adolescents. For example, in the mentioned study by Kautiainen et al. [12] the association of computer use for emailing, writing and surfing the internet and overweight was statistically significant only in the girls. Furthermore, Falbe et al. [8] found longitudinal associations between digital game playing and increased BMI in girls only. However, in the longitudinal study by Altenberg et al. [13] computer time predicted changes in BMI in the boys, and not in the girls.

Using the internet for communication and social networking has been considered to be a more common activity among females compared to males [3, 29]. In our study population, the reported amount of leisure time spent on emailing and chatting was about the same in both sexes, which is in accordance with Swedish internet statistics from 2013 [3]. But, there are apparent gender differences in time spent on computer gaming. A literature review on gender differences in online gaming [6] showed many similarities in males and females’ motivations to play online games, but males tend to play more action and simulation games, while females play more logic and skills training games. We have no information about game content in our study.

### Possible mechanisms

But how is it that women gamers in particular seemed to be vulnerable to weight gain in our study? One of the potential mechanisms for weight gain in connection to screen time is the sedentary nature of the activity, i.e. sitting behaviors and low energy expenditure. In Vandelanotte et al. [19], time spent by the computer was associated also with increased time spent on other sedentary activities. Interestingly, on the national public health level in Sweden, the increased use of computers the past decades, and thus the inferred increase of sedentary activities, has been paralleled with an actual increase in reported leisure time physical activity [30]. In our data, the high gamers reported lower levels of physical activity compared to the others. While this also applied to the men, it was especially pronounced among the women gamers. However, the associations between time spent on gaming and overweight were significant even after adjusting for level of physical activity. Moreover, an additional stratified regression analysis showed that gaming was associated with increased change in BMI in all levels of physical activity in the women (data not shown). It is plausible that regular physical exercise a few times per week does not compensate for physical inactivity the rest of the week. It is also possible that physical inactivity by the computer is especially detrimental to women. In a study by Scheers et al. [31] showing associations between BMI and decreased physical activity levels in both men and women (measured with actigraphs), the duration of sedentary bouts and number of breaks in sedentary time was related to BMI mainly in women. It is possible that our female gamers are subject to longer bouts of inactivity than the men even if the total time by the computer is the same. In this line of reasoning, a possible explanation for why the time spent on emailing/chatting was not to the same extent related to overweight, could be that these communication activities not necessarily imply long durations of physical inactivity even if the total time spent is the same, and thus, maybe a higher energy expenditure.

On the other hand, there is evidence that diet is the most important factor for weight gain [32, 33]. Screen time has been associated with a less healthy diet including higher consumption of energy dense snacks and drinks and lower consumption of fruits and vegetables, among children, adolescents and adults [15, 34, 35]. Computer gaming has been suggested to entail a lower energy intake in combination with a slightly higher energy expenditure, compared to TV viewing, as both hands may be busy using the controls (e.g. [16]). Also, TV viewing to a larger extent implies exposure to advertisements for fat and sugary foods [15, 16, 36]. But the question is then if there are gender differences in energy intake while at the computer? Unfortunately, we have no data on diet in our study. However, there is some evidence that gender differences may exist in this regard; in the systematic review by Pearson et al. [34] of dietary intake and sedentary behavior, about half of those studies that examined gender differences observed them. The conclusion was that the associations between sedentary behavior and diet were more consistent for females than for males. Thus, diet may be an underlying issue in our population.

Sleep is another possible mediator between computer use and overweight, for example shown by Arora et al. [14]. Screen activities may interfere with sleep [37–39] and short sleep is associated with overweight and obesity [40–42]. There were no major differences in reported sleep between the men and women in our study. We have previously reported [43] that the “high email/chatters” as well as the “high gamers” of both sexes more often reported having sleep problems than the “low” groups (prevalence ratios in the range of 1.3–1.4 after adjusting for relationship status, educational level, and occupation). Moreover, the “high email/chatters” of both sexes had an increased prospective risk to have developed sleep problems after one year (prospective PRs 1.9 and 1.7, for men and women, respectively) [37]. However, there may be gender differences in the association between sleep and weight [41]. Self-reported short sleep duration was associated with increased weight in the men but not in the women in a study of young adults by Meyer et al. [42], while self-reported sleep problems (falling asleep or staying asleep) was valid for the women and not for the men.

Another aspect that needs to be addressed is that computer gaming is much less common among women. This raises the question if the women gamers possibly are a more select group than the male gamers. Apart from the fact that women play less than men, women seem to take up gaming later in life, and the average female (online) gamer is older than the male [6]. Due to the limited age span of our study population we could not investigate age-related gender differences. As mentioned earlier, the female gamers seemed to have lower levels of physical activity. The high gaming women also reported being subject to lower social support in private life, and thirty percent were neither in work nor in school. In a previous study in the same cohort, we found that the female computer gamers more often reported stress and depressive symptoms [43], and they had a prospective risk of developing depressive symptoms [37]. There is a reciprocal relationship between obesity and depression, i.e. obesity can lead to depression, but depression can also lead to obesity in some women [44]. Altogether, these women may be subject to several health-related risk factors.

It should also be considered that it is possible that a more detailed categorization of the exposure variables, with higher cut-offs than >2 h per day, may have indicated intensive gaming to be a risk factor for overweight development also in the men.

### Methodological considerations

The strengths of this study include the prospective design with follow-ups after one and five years, and a fairly large study group from a population-based sample. However, there are also several limitations that should be considered. All variables except for age and sex are based on self-reported data. Self-reported BMI is known to be underestimated, mostly because of the underreporting of weight [45, 46], and this may bias the results in unknown ways. Another concern in relation to BMI is that height was only asked for in the baseline questionnaire. BMIs could be overestimated at the follow-ups, due to the fact that men may still be growing at the age of 20–24, although, the proportion of men still growing in this group is probably small.

Further, the validity of self-reported computer use may be questioned [47, 48], implying recall difficulties and recall bias. There may also be potential misclassifications of the two main exposure variables because of them not being mutually exclusive: chatting is sometimes part of computer gaming. Both are included in the total computer use variable and some illogical reports are seen in Table 1, which puts light on the limitations of self-reported data. Further, we assessed only two types of leisure time computer use; gaming and communicating, and thus fail to examine other potential leisure time computer activities. These two were chosen because they were the dominating leisure pastimes by the computer that emerged in a qualitative interview study about computer use and potential mental health effects [49]. It should also be pointed out that the data collection started in 2007, which is prior to the broad use of social media applications such as Facebook, Twitter, etc. Moreover, we do not examine gaming and communicating on other devices. It can be questioned if it is relevant to single out computers as a specific exposure, as technological development gives us a variety of devices for similar activities. For example, in the computer gaming reference group (i.e. no computer gaming), there may be participants who are heavy gamers but on other types of consoles, which hypothetically would entail the same type of consequences. Thus, this type of “misclassification” may dilute effects. Furthermore, the baseline data was collected before the widespread use of smartphones and the mobile internet, but there are probably participants who handled emails and chatting via a mobile phone at the time of the data collection. This may also be a source of misclassification. In order to keep up with developments, the questionnaire items were changed from only concerning computer use to also including smartphones and tablets at the 5-year follow-up in 2012. Although this is a reasonable modernization, it limits the comparability of the exposure data from baseline to the 5 year follow-up. Regardless, it was inevitable for the present study to fail to take into account the diversity of technologies and applications that may today be present. Exposure assessment that is applicable in longitudinal studies is a challenge when studying the modern technologies. Future studies could probably be strengthened by the use of objective exposure measures. It also seems relevant with more detailed assessments of the factors involved in the potential mechanisms. Among the limitations of our study, is the lack of information about, for example, time spent on other sedentary activities, and diet.

The study group confers some limitations due to selection biases. There was a low response rate at baseline and attrition to the follow-ups. A healthy selection can be expected. Women were overrepresented in the study group, and a non-respondent analysis at baseline showed that also native Swedes were overrepresented [22]. Although we adjusted for some demographic and lifestyle factors, only baseline data were used for this, and there may be other confounding factors that are unaccounted for. For example, socioeconomic position (SEP) has been seen to have an inverse relationship with overweight in several European countries, including Sweden [50]. We lack a relevant marker for SEP in the study. Educational level, which is often used as a marker for SEP, was considered to be of low relevance in this young adult group. Naturally, in the course of the five years of study, educational levels increased, and a higher proportion of the participants worked, rather than studied, at the 5 year follow-up. Finally, carrying out separate analyses for men and women eliminated confounding due to sex, but the low response-rate, especially among the men, suggests that caution should be used when generalizing the results to a general population of (Swedish) young adults.

### Implications

We have identified a new risk group for overweight development: young adult female computer gamers. The estimated additional increase of 1.33 BMI-units in the 5-year follow-up period for those who gamed >2 h per day at baseline, would for a woman of average height and weight in the cohort, correspond to an additional weight gain of 3.7 kg. The results should warrant attention for further research to confirm or mitigate the results. As overweight and obesity bring about negative consequences for health and quality of life, especially in females [51, 52], this could be an important group to target for public health preventive strategies. It seems necessary to investigate more closely which mechanisms can be at play in the suggested weight development, in order to develop relevant interventions and direct the right actions.

## Conclusions

We have identified a risk group for overweight development: young adult female computer gamers. Leisure time computer gaming was a risk factor for overweight development in the young women even after adjusting for demographic and lifestyle factors including physical activity, but not in the men. There were no clear prospective associations between computer use for communication (emailing and chatting) and overweight in either sex.

## Abbreviations

CI: Confidence interval
BMI: Body mass index
OR: Odds ratio
PR: Prevalence ratio
SEP: Socioeconomic position
